# Rapid, open-source, and automated quantification of the head twitch response in C57BL/6J mice using DeepLabCut and Simple Behavioral Analysis

**DOI:** 10.1101/2025.04.28.650242

**Published:** 2025-05-01

**Authors:** Alexander D. Maitland, Nicholas R. Gonzalez, Donna Walther, Francisco Pereira, Michael H. Baumann, Grant C. Glatfelter

## Abstract

Serotonergic psychedelics induce the head twitch response (HTR) in mice, an index of serotonin (5-HT) 2A receptor (5-HT2A) agonism and a behavioral proxy for psychedelic effects in humans. Existing methods for detecting HTRs include time-consuming visual scoring, invasive magnetometer-based approaches, and analysis of videos using semi- automated commercial software. Here, we present a new automated approach for quantifying HTRs using the open-source machine learning-based toolkits, DeepLabCut (DLC) and Simple Behavioral Analysis (SimBA). First, pose estimation DLC models were trained to predict X,Y coordinates of 13 body parts of C57BL/6J mice using historical experimental videos of HTRs induced by various psychedelic drugs and experimental conditions. Next, a non-overlapping set of historical experimental videos was analyzed and used to train SimBA random forest behavioral classifiers to predict the presence of the HTR. The DLC+SimBA approach was then validated using a separate subset of visually scored videos. DLC+SimBA model performance was assessed at different video frame rates (120, 60, 30 frames per second or fps) and resolutions (50%, 25%, 12.5%). Our results indicate that HTRs can be quantified accurately at 120 fps and 50% resolution (precision = 95.45, recall = 95.56, F1 = 95.51) or at lower frame rates (i.e., 60 fps and 50% resolution, precision = 91.00, recall = 86.23, F1 = 88.55). The best performing DLC+SimBA model combination was deployed to evaluate the effects of bufotenine, a tryptamine derivative with uncharacterized potency and efficacy in the HTR paradigm. Interestingly, bufotenine only induced elevated HTRs (ED50 = 0.99 mg/kg, max counts = 24) when serotonin 1A receptors (5-HT1A) were pharmacologically blocked. HTR scores for a subset of 21 videos from bufotenine experiments were strongly correlated across DLC+SimBA, visual review, and semi- automated software detection methods ( *r* = 0.98 -0.99). In summary, the DLC+SimBA approach represents a rapid and accurate new method to detect HTRs from experimental video recordings using open-source toolkits.

**TOC Graphic:**

## Full Text Availability

The license terms selected by the author(s) for this preprint version do not permit archiving in PMC. The full text is available from the preprint server.

